# Genes sharing the protein family domain decrease the performance of classification with RNA-seq genomic signatures

**DOI:** 10.1186/s13062-018-0205-x

**Published:** 2018-02-21

**Authors:** Anna Leśniewska, Joanna Zyprych-Walczak, Alicja Szabelska-Beręsewicz, Michal J. Okoniewski

**Affiliations:** 1 Department of Computer Science, Poznan University of Technology, Piotrowo 2, Poznan, 60-965 Poland; 2 Department of Mathematical and Statistical Methods, Poznan University of Life Sciences, Poznan, 60-637 Poland; 3 Scientific IT Services, ETH Zurich, Weinbergstrasse 11, Zürich, 8092 Switzerland

**Keywords:** RNA sequencing, Data Analysis, Machine Learning, Statistics, Protein domains, Genomic signatures, Biomarkers

## Abstract

**Background:**

The experience with running various types of classification on the CAMDA neuroblastoma dataset have led us to the conclusion that the results are not always obvious and may differ depending on type of analysis and selection of genes used for classification. This paper aims in pointing out several factors that may influence the downstream machine learning analysis. In particular those factors are: type of the primary analysis, type of the classifier and increased correlation between the genes sharing a protein domain. They influence the analysis directly, but also interplay between them may be important.

We have compiled the gene-domain database and used it for analysis to see the differences between the genes that share a domain versus the rest of the genes in the datasets.

**Results:**

The major findings are:

- pairs of genes that share a domain have an increased Spearman’s correlation coefficients of counts;
- genes sharing a domain are expected to have a lower predictive power due to increased correlation. For most of the cases it can be seen with the higher number of misclassified samples;
- classifiers performance may vary depending on a method, still in most cases using genes sharing a domain in the training set results in a higher misclassification rate;
- increased correlation in genes sharing a domain results most often in worse performance of the classifiers regardless of the primary analysis tools used, even if the primary analysis alignment yield varies.

**Conclusions:**

The effect of sharing a domain is likely more a results of real biological co-expression than just sequence similarity and artifacts of mapping and counting. Still, this is more difficult to conclude and needs further research.

The effect is interesting itself, but we also point out some practical aspects in which it may influence the RNA sequencing analysis and RNA biomarker use. In particular it means that a gene signature biomarker set build out of RNA-sequencing results should be depleted for genes sharing common domains. It may cause to perform better when applying classification.

**Reviewers:**

This article was reviewed by Dimitar Vassiliev and Susmita Datta.

## Background

The CAMDA data analysis challenge neuroblastoma dataset includes disease phenotype classes assigned to samples that can be attempted to be verified by running a classification with cross-validation. However, our experience with machine learning approaches on this dataset has proven that this type of task is not particularly trivial. The parameters of classification such as number of misclassified samples were varying between the methods applied.

This is why we decided to look deeper into the factors that make difficult using RNA sequencing as a biomarker input for machine learning techniques. This paper describes the experience with three major sources of bias and uncertainty in such analyses. In particular we investigated the impact of expression similarities and correlations for genes sharing a protein domain. In addition, the interplay between a primary analysis (alignment) and types of classifier is taken into account.

We do not intend to present just a negative results paper stating that the classification task is difficult in reaching the “biological truth”. Apart from showing difficulties in the analyses, we point out good practices that may be leading to better practical utility of classification based upon RNA sequencing.

This is also why we decided to go beyond just the CAMDA dataset. Three public datasets similar in size and content to the CAMDA one have been used to make the outcome more comprehensible.

### Correlation-based approaches in RNA sequencing

Many of the methods of data analysis in transcriptomics use specific measures for genes co-expression. One of the most obvious approaches is using a correlation coefficient. It is in fact the basis for popular heatmaps and hierarchical clustering of measured samples. However, as pointed out in the study [1] the positive correlations between the transcriptomics measurements may be an effect of real biological co-expression as well as artefactual correlation due to the technology specific issues. It is practically not possible to fully distinguish the increased correlation from both of the reasons. The study [1] has proven that in the Affymetrix techonology the increased correlation is seen for probesets that share genes with the same sequence.

### Lack of an ideal database of nucleotide-level similarity in domains

In this analysis we propose an approach that is focused on gene structure and sequence composition in context of genome-wide analysis concerning the influence of protein domains, using the information from the PFAM database [2]. The domains described in PFAM are the results of aminoacid-level analysis of sequences, thus not all the protein domain may have enough similarities on the nucleotide level of mRNA. Still, we use it as an initial approximation for sequence similarity, as creating a similar nucleotide database may be non-trivial, eg. the databse RFAM [3] includes only domains in non-coding sequences.

## Methods

### Database of genes and domains

**Fig. 1:**
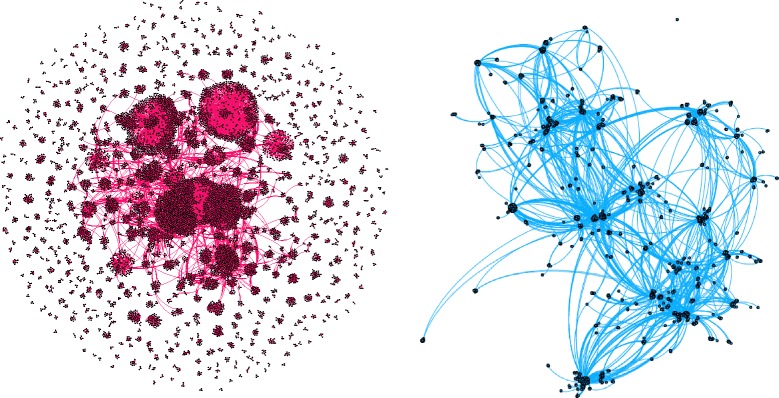
Graphs visualized in Gephi, depicting genes interconnected with domains. Left - the global picture, right - a single disconnected sub-graph. It shows that the interconnection of domains in the genes are not regular and trivial

As the first step in the analysis the global table of protein family domains and genes in which they are located was built from annotation databases. Appropriate database joins have been performed on the genomic coordinates of genes (AceView for CAMDA dataset or Ensembl) and domains from Pfam. The data may be interpreted as a graph where the nodes are genes and domains. The graph consists of gene-domain-gene motifs, as a gene is connected with another gene always via a domain and vice versa. This builds the structural “galaxies” of gene families interconnected with domains (see Fig. 1). The graphs in 1 were created using Gephi tool (ver.0.9.1) [4].

### Datasets analyzed

Additional analysis is using three different datasets from NCBI Gene Expression Omnibus (GEO) public database [5] with the data series accession numbers GSE22260, GSE50760 and GSE87340. All of them contain human RNA-seq data for cancer-related studies.

- Dataset **GSE50760** [6]. Dataset includes RNA-seq data of 54 samples (normal colon, primary CRC, and liver metastasis) were generated from 18 CRC patients.
- Dataset **GSE22260** [7]. Dataset includes RNA-seq data of 20 samples prostate cancer tumors and 10 samples matched normal tissues.
- Dataset **GSE87340** [8]). Dataset includes RNA-seq data of 54 samples, 27 pairs of tumor and normal tissues from lung adenocarcinoma patients. Since there were samples with paired-end and single end reads, we have only used the subset of 44 samples out of 54 with single-end reads.

### RNA sequencing data processing

Data transformation and processing was performed by the following RNA-seq primary analysis workflow: SRA Toolkit (ver.2.8.2) was used to achieve the raw data in fastq format. Three different mappers (Hisat2 (ver.2.1.0) [9], Subread (ver.1.5.2) [10], Star (ver.2.5) [11]) have been used to align the reads to the reference human genome (GRCh38/hg38). Mappers were used with their default settings. The reference genome index for each mapper has been built with the internal tools based on the reference human genome. SAMtools (ver.1.2) [12] and featureCounts function [13] from package Subread (ver.1.5.2) [10] were used to perform gene counting. Differential gene expression was identified from gene-level read counts using edgeR [14].

### Co-expression of genes sharing a domain

The analysis included calculating co-expression coefficients for genes that share a structural domain. Gene expression values for different samples can be represented as a vector. Thus calculating the co-expression measure between a pair of genes is the same as calculating the selected measure for two vectors of numbers. It is assumed that count data follows negative binomial distribution. That is why we checked one of the most commonly used co-expression measures - Spearman’s rank correlation coefficient, following the method from [1]. This measume is a nonparametric (distribution-free) rank statistic that allows to calculate correlation for non-Gaussian distributions. The distributions of correlation have been generated for the gene pairs sharing a domain and for a random gene pairs without a domain.

In addition, machine learning approaches have been used for finding the effectiveness of prediction of some differentially expressed genes. First, the differential expression was performed with edgeR approach [14]. We choose as a differentially expressed genes all the genes with the significance level *α*=0.05.

### Machine learing approaches - classification of samples

Then, in this set, we looked for the domain that is connected with the biggest number of genes. Next, we calculated the classification error taking into account those chosen genes and as the opposite - the second subset consisted of genes sharing no domains. In each dataset the classification attribute was the sample group division from the published experiments. As the variables in the classifiers count data tables of the chosen genes connected with one domain or the top of differentially expressed genes without domains were used. The number of genes that was taken for the machine learning was limited with half of the number of samples in the experiments, to avoid overtraining (Hughes phenomenon) [15]. We trained the classifiers based on these variables to find if the sample matches the particular clinical phenotype group. We used the following classifiers: k-nearest neighbor [16], support vector machine [17], the neural network [18] and random forest [19]. All of these classifiers are included in the MLInterfaces R package [20]. This package unifies the Bioconductor approached to the classification, thuse we treated it as a “golden standard” in the area. 5 fold cross-validation was used to calculate prediction errors counted as misclassification of samples. An alternative, in particular in practical solutions, would be using ensemble or ranked classifiers, eg as described in [21], still in this study we intend to test mainly the performance of well-known general purpose classifiers to point out possible artifacts with domain-associated genes in the input data. All the analyses have been carried out using R v3.4.0 and BioConductor v3.4.

## Results and Discussion

### Initial results with the CAMDA dataset

**Fig. 2:**
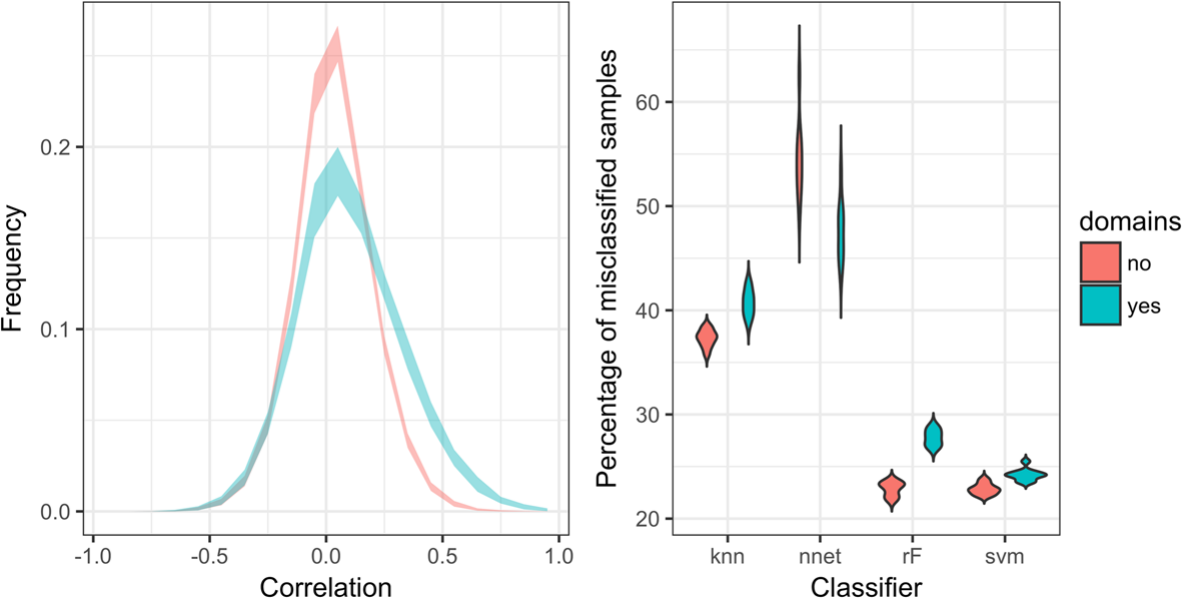
Spearman’s correlation distribution and violinplots of percentage of misclassified samples for genes with and without domains in CAMDA neuroblastoma dataset. On the left the red color is for the histogram-based distribution of Spearman’s correlation coefficient for a random selection of gene pairs without domains. Green color stands for Spearman’s correlation coefficient for the genes that share a PFAM domains (database built with AceView genes). Shades in the line are ranges from 100 simulations of the distribution. On the right there is violin plot of percentage of misclassified samples for 4 classifiers based on DEG with and without domains. Total number of samples in dataset was 302

For the CAMDA neuroblastoma dataset the Spearman’s correlation distribution have shown a shift towards positive values for the gene pairs linked by a domain. Only in the case of neural networks the classification with the genes sharing domain was better than without (see Fig. 2). Those results have been the direct motivation to test this approach with other datasets.

### Properties of datasets mapped to the reference genome

**Fig. 3:**
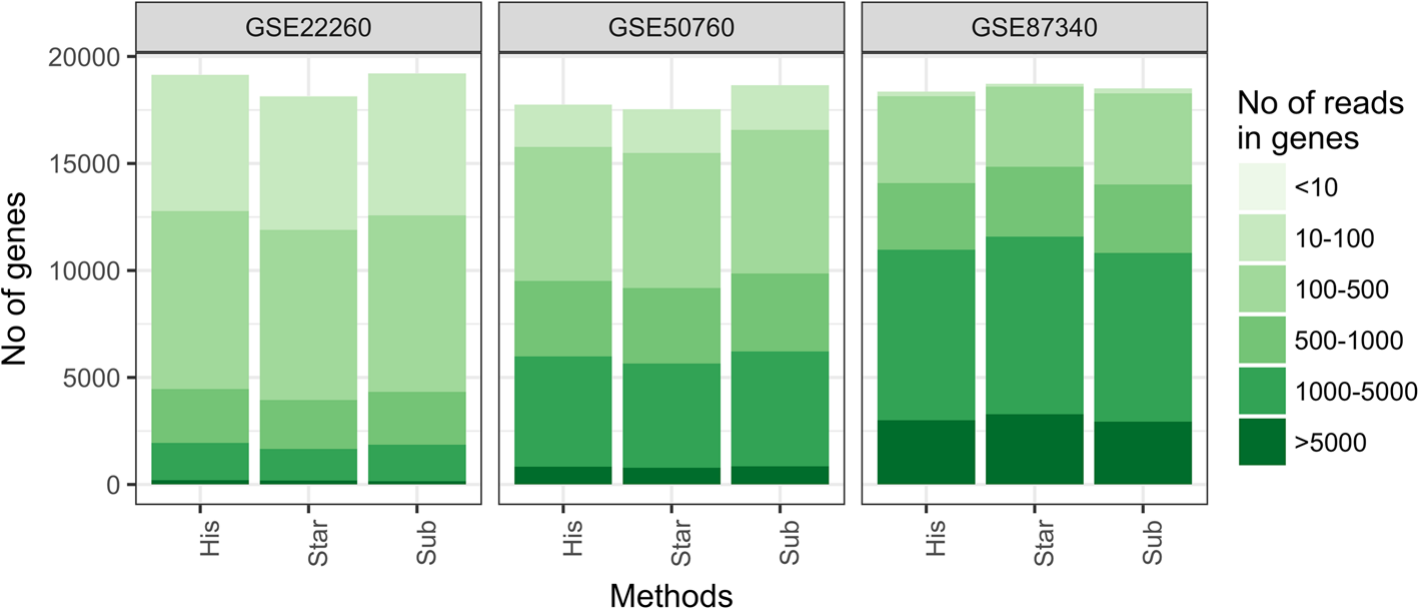
Division of genes based on number of reads aligned to those genes. Barplots of the number of genes with the division of number of reads assigned for the genes for three datasets from the NCBI GEO public database, aligned with three different mappers (Hisat2, Star, Subread) were generated. Colors in barplots mean the ranges of number of reads that are aligned to the genes

The datasets have been aligned to the reference genome using three different mappers in order to see the influence of the alignment software. The distribution of reads abundance in genes shows that no particular mapper proves to be clearly superior. Also the three datasets differ significantly in the reads abundance in genes (see Fig. 3).

### Standard differential expression analysis

In all the datasets a differential expression analysis has been performed with edgeR. Typically, almost half of the significantly expressed genes are those that have a PFAM domain (see Table 1).

**Table 1:** Number of differentially expressed genes (DEG) with and without domains for considered datasets and mappers

Mapers	No of DEG	Datasets		
		GSE22260	GSE50760	GSE87340
Hisat	Total	359	7182	11048
	With/no domains	245 / 114	5141 / 2041	7839 / 3209
Star	Total	430	7264	11619
	With/no domains	271 / 159	5165 / 2055	7985 / 3634
Subread	Total	579	7918	11402
	With/no domains	369 / 210	5350 / 2568	8029 / 3373

For each dataset and mapper the number of total number of DEG, as well as number of DEG with and without domains was calculated. In each case there were more DEG with domains

### Analysis using the knowledge of shared domains

For the database integration done with Pfam and AceView, there are 20566 genes that share a domain, and 12666 genes without a domain. For analogous Ensembl joins there are 16923 genes with the domain and 41069 without.

**Fig. 4:**
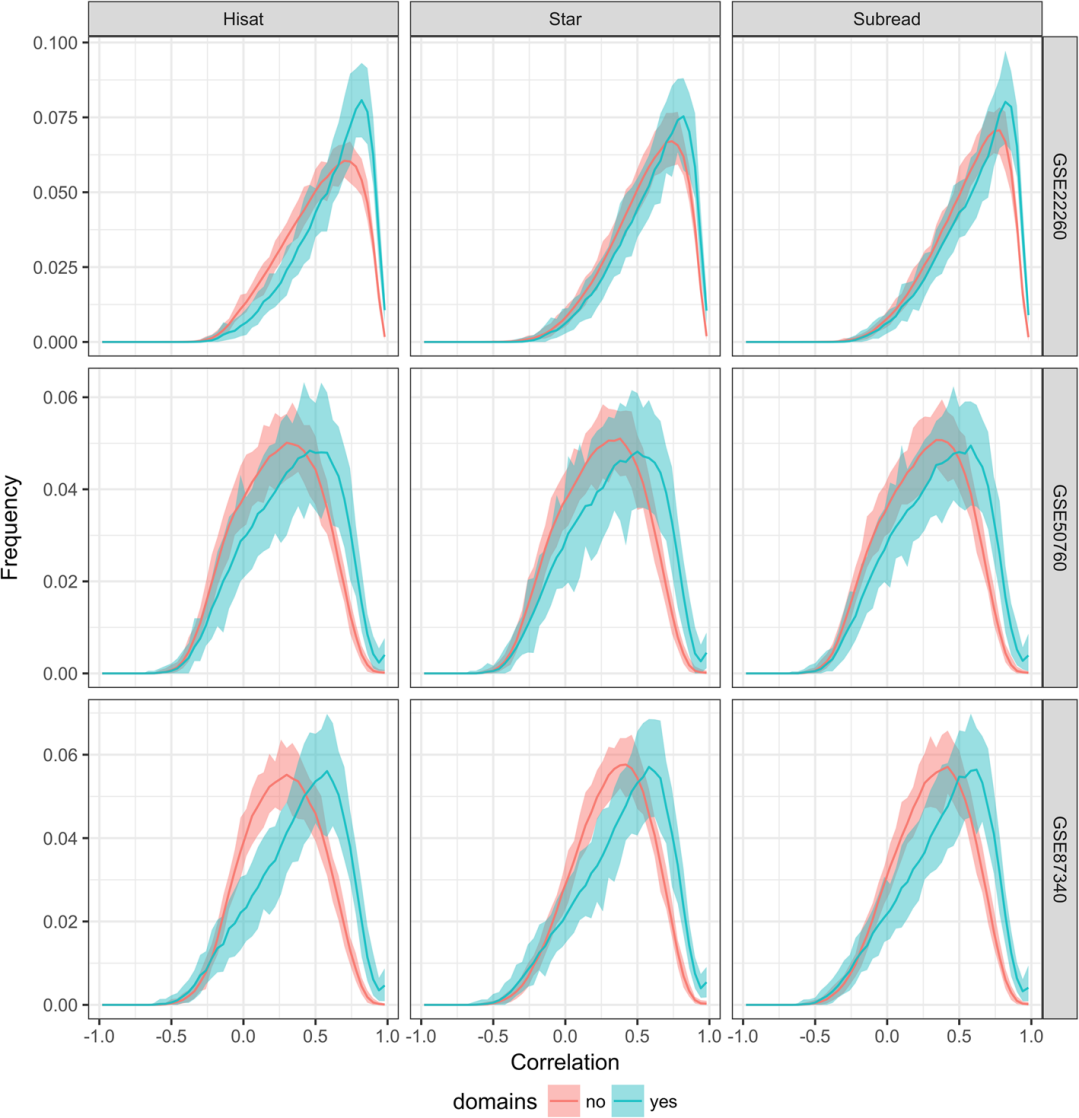
Spearman’s correlation distribution for the pairs of genes with and without domains. Red color is reserved for the histogram-based distribution of a correlation between random selection of 25000 gene pairs without domains. Green color is connected with Spearman’s correlation coefficient for 25000 genes that share a PFAM domains. Lines in the middle are the mean distributions of correlation based on 100 simulations of the choice of genes. Shades in lines signify minimum and maximum values based on 100 simulations. Genes with domains have shifted correlation to the right

We have calculated Spearman’s correlation coefficient between the expression values of genes that share the same domain and between the expression values of genes that do not share any domain. Figure 4 depicts the histogram-based distributions of correlation coefficients between the log value of counts for 25000 randomly chosen pairs of genes that share the same domains (green) or do not share any domain (red).

The increased correlation is visible in all the cases. In the Affymetrix technology such phenomenon was explained partly by the artifacts of sequence similarity, partly by a real biological co-expression [1]. In RNA-seq one can try to distinguish between those two types of effects on correlation by counting or not the multiple mapping reads, eg using featureCount [10]. Typically, the count tables of not multiple mapped genes include smaller numbers of reads. We have studied the differences between the counting with and without multiple mapping reads, but the results were not conclusive. There is some “signal propagation” between the genes sharing a domain in the case of multiple mapping, but it was hard to calculate that it has significant effect on the correlation increase. The effect of increased correlation exists, thus it has to be assumed that it is a mixture of biological co-expression and multiple mapping of reads to the similar sequences in domains.

### Influence of genes with domains on the classification outcome

The result of RNA-seq experiments are the datasets describing the expression of thousands of genes simultaneously. This explains the increase of the computational complexity involved in the classification process and has an adverse effect on the estimation of the prediction. In this part of our investigations we wanted to determine what is the prediction error in the case of classification. The gene selection process can help to obtain a subset of genes that can be used to distinguish different sample classes, often called a genomic signature. Therefore, it is important to carry out this step of analysis as efficiently as possible.

The idea was to take into account the correlation structure of the genes in the selection process. We used the assumption from [22] proven additionally in the [23] that genes that are highly correlated with one to another, often belong to the same metabolic pathways or perform similar functions in the cells. Similar point in the context of genomic variant data was made in the study [24]. Thus in the classification process one should avoid the selection of highly correlated genes because they do not contribute with much additional information to the classification [25] and also generate similar prediction errors in the process of discriminant analysis [26]. Therefore we used two subsets of significant genes: with and without domains.

**Fig. 5:**
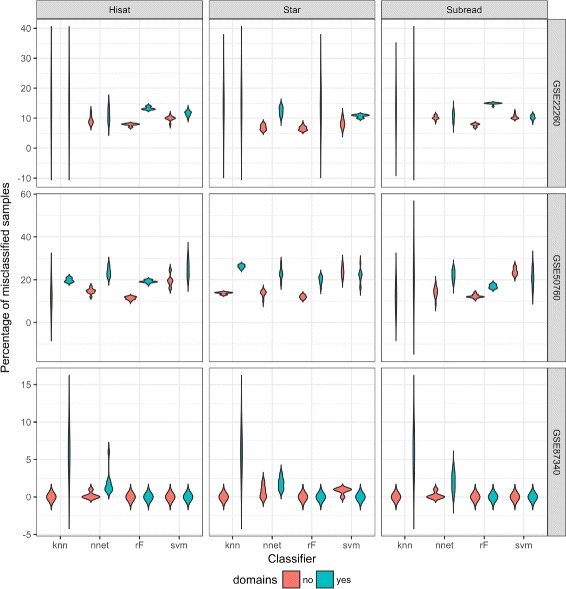
Violinplot of misclassified samples for 4 classifiers based on DEGs with and without domains. From the differentially expressed genes with the significance level *α*=0.05 we choose two subsets: the first one was the genes that share one particular domain (with the biggest number of genes connected to this domain) and the second was the genes that share no domain. Validation was performed with 5 fold cross-validation. Percentages of misclassified samples are mostly lower for the cases where genes with no domains are taken into account

Having the confirmation that correlation for genes with domains was shifted to the right compared to the ones without any domain, we wanted to check what will be the prediction efficiency. From the results of machine learning most classifiers performed better when DEG without domains were used as variables (see Fig. 5). The knn classifier in the case of genes with domain has a high variety for most of the datasets and mappers. Neural network and random forest classifiers in the case of genes without domains result with the lowest percentages of the misclassification. In most of the cases, the classifiers trained using genes with domains had on average more misclassified samples. Only in the case of SVM as a classifier, in particular with STAR or Subread mapping, the effect was slightly opposite - genes with domains performed a bit better with classification. Combination of SVM and Hisat2 mapping was giving more misclassification with domains.

## Conclusions

The main direct conclusion from the validation of machine learning techniques based on two cases is that we get lower percentages of misclassified samples for the case where genes with no domains are taken into account. Using a genomic signature with genes sharing a domain leeds most often to worse and less informative results of classification. The way out can be eg. drawing a graph of domain connections for the genomic signature such as in 1 and replacing some of the domain-connected genes with subsequent significant ones. Another good practice that can be suggested is checking various types of classifiers - as there is no obviously superior one. In addition, the classifiers perform differently for genes sharing a domain. Like other aspects of RNA sequencing analysis results, it is very much dataset dependent.

## Reviewers’ comments

### Reviewer’s report 1: Dimitar Vassilev, Faculty of Mathematics and Informatics, Sofia University, Bulgaria

The submitted manuscript is result of interesting data analysis research approach. It is valuable and has some obvious merits in particular for providing a platform for validation of the methods used for classification of genes sharing protein family domains. From a methodological point of view it is obvious that authors applied a decent arsenal of statistical methods and machine learning procedures. The English language is at a decent level and a possible minor stylistic improvement will be very helpful to the manuscript. The results of the study reveal the influence of some studied factors on the classification of the studied genes. Authors discussed that by the lower predictive power of the genes sharing domain. This is related also to the right choice of the classifiers, which performance may vary depending on the method applied. The “noise” in genes classification also is related to the increased values of the correlation of counts. I think that the methodological side of the submitted manuscript is logical and has enough diverse approaches and methods for cross validation of the results and confirming the authors these of the work. Although I have some remarks concerning the methodology constructed by authors.

1. Correlations are so-called second-moment estimators and they have certain error levels. The acceptance of the Spearman rank correlation is not well defined as a choice among other correlation methods as Pearson, Kendall, etc. This could throw more light on the explanation of the behaviour of the subsequently used classifiers.

2. In this line few words about the initial data concerning the distribution will be useful and a eloquent explanation why the Spearman correlation was chosen

3. The machine learning methods vary by their nature and it is difficult to choose the correct method. The the choice and a subsequent comparison of the used four machine learning methods should be additionally explained and related to the variation of the classifiers.

4. The machine learning classification approaches used by authors have opened some methodological questions which are more related to the methods for preprocessing of the data and the direct use of deep learning could not lead to desired results. I will suggest the deep learning methodology suggestions made at the end of the conclusions to be removed.

**Authors’ response:**

*1. and 2. Although Pearson’s correlation coefficient could be effective as a similarity measure for gene expression data [* 27 *] the main drawback of Pearson’s correlation coefficient is that it assumes an approximate Gaussian distribution and may not be robust for non-Gaussian distributions [* 28 *]. We are assuming that read counts follow negative binomial distribution. To address this, Spearman’s rank-order correlation coefficient has been suggested in literature as one of the alternative similarity measures [* 29 *]. Spearman’s rank correlation coefficient is a nonparametric (distribution-free) rank statistic. It is a measure of a monotone association that is used without making any assumptions about the frequency distribution of the variables.*

*3. It was one of the issues that the paper attempts to point out: that the machine learning methods vary in their purpose, characteristics and performance on specific dataset, but what agrees is that they perform generally better on genes that do not share common domains. The selection of machine learning methods thus was intended to include most typical and popular ones so we decided on those used in the package MLInterfaces [* 20 *], which is a sort of unifying approach for classifiers. The explanation in the Methods section was expanded accordingly.*

*4. The deep learning reference was too far-fetched indeed, so we have removed it following the Reviewer advice.*

### Reviewer’s report 2: Susmita Datta, Department of Biostatistics, University of Florida, Gainesville, USA

In this work authors analyzed the Neuroblastoma CAMDA challenge data to identify samples with genomic biomarkers using RNA-deq data. In doing so, they realized that the classification results vary across different tuning parameters of a classification algorithm. Hence they analyzed three more GEO data in order to reconfirm their findings about this fact of classification. In order to do that, they have realized that the genes sharing common domains are correlated and moreover, classifying samples with the correlated genes resulted poorer classification accuracy. Moreover, the classification accuracy depended on the tuning parameters of the algorithms.

Although the results are interesting for these datasets, in general, these results have been shown before. The basic idea for using group LASSO and fused support vector machine (Rapaport et al., 2008) where a fused penalty is added enforcing similar weights on correlated features provides better classification compared to just LASSO. The result of the difference in classification accuracy, using different classification algorithms and different tuning parameters have been widely noted and Datta, Pihur and Datta (BMC Bioinformatics, 2010) provided a solution to the problem by proposing an adaptive optimal ensemble classifier via bagging and rank aggregation. This optimal ensemble classifier performs at least as best as the best classifier within a set of given classifiers with different tuning parameters. Authors must provide the references and acknowledge these established results and possibly use them in this context. Nevertheless, the findings of this manuscript are correct and noteworthy.

**Authors’ response:**

*The issue of classifier performance on genomic is indeed described in a number of papers as it addresses one of the central issues in practical use of genomics results e.g. in precision medicine, namely the genomic signatures. The fact that correlating features influence the outcome of classification we have cited using the study on gene expression [* 22 *] tested and extended in the PhD thesis of one of the authors [* 23 *]. The study [* 24 *] is a complementary one with data from genomic variants, so was mentioned appropriately. To some extent our work is also indeed based on the ideas from extensive benchmarking of machine learning algorithms as described in [* 21 *]. This paper provides also the suggestion of using ensemble classifier. Our main point was a warning in the case of using typical classifiers, so we gladly cite this study as a pointer for the readers towards a more sophisticated, but likely efficient solution, while in this study we intend to test mainly the performance of well-known general purpose classifiers to point out possible artifacts with domain-associated genes in the input data.*

## Abbreviations

CAMDA: Critical assesment of massive data analysis - conference and data analysis challenge
DEG: Differentially expressed genes
KNN: k-nearest neighbors algorithm
NNET: Neural network algorithm
PFAM/RFAM: Databases of protein and nucleotide sequence families
RNA: ribonucleic acid
rF: Random forest algorithm
SVM: support vector machine algorithm
